# O-GlcNAcylation increases non-amyloidogenic processing of the amyloid-β precursor protein (APP)

**DOI:** 10.1186/1750-1326-8-S1-P21

**Published:** 2013-09-13

**Authors:** Kristin Jacobsen, Kerstin Iverfeldt

**Affiliations:** 1 Stockholm University, Stockholm, Sweden

## Background

The amyloid-β precursor protein (APP) has been extensively studied, due to its role in Alzheimer’s disease (AD). Sequential proteolytic processing of APP, catalyzed by β- and γ-secretase, generates the neurotoxic peptide amyloid-β (Aβ), which accumulates in the brain and cause progressive neurodegeneration. However, APP is mainly processed through another pathway, where APP is cleaved by α- and γ-secretase, generating the secreted sAPPα fragment. Stimulation of α-secretase processing of APP constitutes an important therapeutical strategy, not only since it precludes the formation of Aβ, but also because the sAPPα fragment has been shown to have neuroprotective properties [1]. APP was the first plasma membrane protein shown to be O-GlcNAcylated [2], a dynamic post-translational modification involving the attachment of β-N-acetylglucosamine (GlcNAc) catalyzed by O-GlcNAc transferase and O-GlcNAcase. However, the consequences of APP O-GlcNAcylation have so far not been investigated.

## Material and methods

We have used siRNA and pharmacological inhibitors directed against O-GlcNAcase and O-GlcNAc transferase to determine these enzymes regulate O-GlcNAcylation of APP. O-GlcNacylated proteins were immunioprecipiated from cell-lysates using an O-GlcNAc antibody, and APP was then detected by western blot using. The levels of sAPPα and Aβ in the cell medium were analyzed using western blot and ELISA. The studies were mainly preformed in human SH-SY5Y neuroblastoma cells, but other cell-lines were used as well as a comparison.

## Results

Here, we show that O-GlcNAcase and O-GlcNAc transferase regulate the level of APP that is immunoprecipitated with O-GlcNAc antibody in human neuroblastoma SH-SY5Y cells. We also show that O-GlcNAcylation increases α-secretase processing, resulting in increased levels of the neuroprotective sAPPα fragment and decreased Aβ secretion. The proteolytic processing of the APP homologue, APLP2, remained unchanged in response to increased O-GlcNAcylation. Furthermore, the effect of O-GlcNacylation on APP processing seems to be specific for neuron-like cells, since the levels of sAPPα from the human embryonic kidney cell-line HEK293 remains unchanged in response to inhibition of O-GlcNAcase, whereas the neuroblastoma cell-line SK-N-AS show increased sAPPα levels, but not to the same extent as in SH-SY5Y cells.

## Conclusions

We conclude that increased O-GlcNacylation selectively affects APP processing in neuro-like cells. Our results implicate O-GlcNAcylation as a potential therapeutic target for Alzheimer’s disease

## References

[B1] Mattson MP Cheng B Culwell AR Esch FS Lieberburg I Rydel RE Evidence for excitoprotective and intraneuronal calcium-regulating roles for secreted forms of the β-amyloid precursor protein Neuron 1993 10 243 254 10.1016/0896-6273(93)90315-I 8094963

[B2] Griffith LS Mathes M Schmitz B Beta-amyloid precursor protein is modified with O-linked N-acetylglucosamine J Neurosci Res 1995 41 270 278 10.1002/jnr.490410214 7650762

